# Supplementary material for: The Emergence of H7N7 Highly Pathogenic Avian Influenza Virus from Low Pathogenicity Avian Influenza Virus Using an in ovo Embryo Culture Model
Source: Viruses. 2020 Aug 21;12(9):920. doi: 10.3390/v12090920 (PMC7552004; doi:10.3390/v12090920)
Supplement: Supplementary file 1 [file viruses-12-00920-s001.zip › viruses-900737-supplementary.pdf]

Supplementary Material

1. Deep Sequencing methods

Viral RNA was extracted (59) without the addition of carrier RNA and either (i) directly sequenced or (ii) PCR-amplified across the CS region. For (i), double-stranded cDNA was prepared using the cDNA synthesis system, according to manufacturer's instructions (Roche, UK). For (ii), RNA was reverse-transcribed into double-stranded cDNA using Uni 12 primer 5'-AGCAAAAGCAGG-3' at a final concentration 5µM and MMLV-RT enzyme (Promega, UK), followed by the FastStart High Fidelity PCR system (Roche, UK) with H7 CS-specific primers (59) modified to contain the Illumina specific overhang sequencing adapters at a final concentration 0.4µM each:
Forward: 5'-**TCGTCGGCAGCGTCAGATGTGTATAAGAGACAG**CGTGCAAGTTTTCTGAGAGG-3' and reverse: 5'-**GTCTCGTGGGCTCGGAGATGTGTATAAGAGACAG**GACCTTCCCATCCATTTTCA-3'.

PCR cycling conditions: 95°C for 2 minutes; followed by 35 cycles of: 95°C for 30 seconds, 50°C for 30 seconds, 72°C for 2 minutes; with a final extension at 72°C for 5 minutes.

A tailored Python script was written to select sequence reads from raw fastq files starting with 'CCCGAAATCCCAAAG' (PEIPK) and ending with

‘AGAGGCCTATTT’ (RGLF). The resulting output report recorded the total number of reads sequenced and the total number of reads that spanned the entire CS based on the defined motifs. A breakdown of all the nucleotide sequences found between the two defined starting and ending motifs were given with the number of reads for each variant and the proportions of each variant found.

2. Establishing background sequencing limitations

RG viruses and pol I HA plasmids were sequenced to establish the frequency of background variants generated by experimental and technical error. Firstly, separate sequencing was done using (i) the H7N7-RG viruses’ RNA, sequenced multiple times, and (ii) from amplicons of the CS region to obtain independent replicates for comparison. The percentage of correct CS reads obtained from sequencing RNA from the RG viruses directly and from sequencing the CS PCR products ranged from 99.08%-100% and 98.79%-99.69% respectively (Table S1). These frequencies were compared to error levels derived from sequencing pol I HA plasmid DNA that was used to generate the RG viruses to determine an overall background error level. The percentage of correct CS reads obtained from sequencing the plasmids directly ranged from 98.08%-99.82% (Table S2). The percentage of correct CS reads obtained from sequencing the CS PCR targeted products had a narrower range between 99.33%-99.75% (Table S2). The maximum error level obtained from sequencing RNA was 0.92% compared to 1.21% from the PCR products. More variation was obtained when sequenced directly from the plasmids (max. 1.92%) compared to sequencing from PCR products derived

from the plasmids (max. 0.67%). The most conservative error level derived, 1.92%, was applied to sequenced samples derived from *in vitro* and *in ovo* passage experiments. The greater depth offered by enriching for the CS region by PCR, without introducing additional sequencing errors above that seen for direct sequencing from plasmids, was preferred for this CS variant analysis.

Table S1. Deep sequence results from H7N7 RG viruses sequenced from RNA or from DNA following CS amplification by PCR. Samples were sequenced up to three times independently.

	RNA			CS PCR		
RG virus sequenced	Total reads	CS reads	Correct CS reads (%)	Total reads	CS reads	Correct CS reads (%)
$H7N7_{SB}$ (1)	2,211,399	168	167 (99.40%)	270,140	254,759	253,688 (99.58%)
$H7N7_{SB}$ (2)	5,899,294	1102	1098 (99.64%)	224,607	213,992	213,319 (99.69%)
$H7N7_{DB}$ (1)	4,128,249	537	537 (100%)	86,514	81,115	80,542 (99.29%)
$H7N7_{DB}$ (2)	8,631,446	3,030	3,013 (99.44%)	78,011	73,306	72,418 (98.79%)
$H7N7_{DB}$ (3)	*nd*	*nd*	*nd*	3,242,056	2,898,069	2,884,787 (99.54%)
$H7N7_{MB}$ (1)	6,800,855	8,797	8,716 (99.08%)	*nd*	*nd*	*nd*

nd; not done

Table S2. Deep sequence results from pol I HA plasmids. Plasmids directly sequenced three or four times, or amplified by PCR targeting the CS sequenced in two independent runs.

Sample	DNA			CS PCR		
	Total reads	CS reads	Correct CS reads (%)	Total reads	CS reads	Correct CS reads (%)
pPol I H7N7 HA_{SB} (1)	18,606,348	893,333	888,843 (99.50%)	213,630	204,587	203,834 (99.63%)
pPol I H7N7 HA_{SB} (2)	371,387	19,506	19,457 (99.75%)	254,220	246,307	245,701 (99.75%)
pPol I H7N7 HA_{SB} (3)	118,685	6,330	6,252 (98.77%)	*nd*	*nd*	*nd*
pPol I H7N7 HA_{SB} (4)	238,977	12,715	12,512 (98.40%)	*nd*	*nd*	*nd*
pPol I H7N7 HA_{DB} (1)	292,564	14,425	14,346 (99.45%)	108,129	104,406	104,026 (99.64%)
pPol I H7N7 HA_{DB} (2)	297,650	14,815	14,788 (99.82%)	83,053	78,633	78,107 (99.33%)
pPol I H7N7 HA_{DB} (3)	247,054	12,608	12,495 (99.10%)	*nd*	*nd*	*nd*
pPol I H7N7 HA_{MB} (1)	168,902	8,230	8,072 (98.08%)	256,161	247,045	245,986 (99.57%)
pPol I H7N7 HA_{MB} (2)	184,109	8,556	8,493 (99.26%)	267,024	257,429	256,150 (99.51%)
pPol I H7N7 HA_{MB} (3)	233,390	10,809	10,719 (99.17%)	*nd*	*nd*	*nd*

nd; not done

Table S3. Cleavage site variant analysis results from $H7N7_{DB}$ E3, E10 and E14 embryo passaged viruses. All samples sequenced from PCR products unless specified.

Sample	Total reads	CS reads	MB reads (%)	DB reads (%)	SB reads (%)	Other reads (%)
**(A) $H7N7_{DB}$ E3**						
Tissue homogenate	770,590	644,580	341,971 (53.10%)	280,420 (43.50%)	8,068 (1.25%)	14,121 (2.15%)
CEF cell supernatant	710,736	587,308	349,490 (59.51%)	221,071 (37.64%)	4,813 (0.82%)	11,934 (2.03%)
Allantoic fluid[a]	816,760	687,744	222,003 (32.28%)	440,088 (63.99%)	9,680 (1.41%)	15,973 (2.32%)
**(B) $H7N7_{DB}$ E10**						
Tissue homogenate	7,904,568	6,541,266	4,529,484 (69.24%)	1,935,289 (29.59%)	0 (0.00%)	76,493 (1.17%)
CEF cell supernatant	721,272	613,267	598,803 (97.64%)	3,147 (0.51%)	0 (0.00%)	11,317 (1.85%)
Allantoic fluid[a]	1,743,746	1,447,041	1,368,801 (94.59%)	48,985 (3.39%)	0 (0.00%)	29,255 (2.02%)
Allantoic fluid[a,b]	4,820,626	1,920	1,794 (93.44%)	58 (3.02%)	0 (0.00%)	68 (3.54%)
**(C) $H7N7_{DB}$ E14**						
FFPE head and membranes	463,370	412,148	109,297 (26.52%)	299,934 (72.77%)	1 (0.00%)	2,916 (0.71%)
FFPE body	1,669,997	1,469,994	1,454,394 (98.94%)	245 (0.02%)	0 (0.00%)	15,355 (1.04%)
**$H7N7_{DB}$ E3** Allantoic fluid	441,963	378,665	522 (0.14%)	361,119 (95.37%)	7,449 (1.97%)	9,575 (2.52%)
**$H7N7_{DB}$ E10** Allantoic fluid	5,745,780	5,078,863	0 (0.00%)	5,057,280 (99.58%)	0 (0.00%)	21,583 (0.42%)

[a]Allantoic fluid after inoculation with CEF supernatant, [b] Sequenced from RNA

Table S4. Cleavage site variant analysis results from $H7N7_{SB}$ embryo passaged viruses. All samples sequenced from PCR products unless specified.

	Sample	Total reads	CS reads	MB reads (%)	DB reads (%)	SB reads (%)	Other reads (%)
**Passage 1: Tissue homogenates**							
**A**	$H7N7_{SB}$ E6	323,943	272,940	65 (0.02%)	1452 (0.53%)	268,777 (98.47%)	2646 (0.98%)
**B**	$H7N7_{SB}$ E12	532,669	457,607	169 (0.04%)	1124 (0.25%)	452,935 (98.98%)	3379 (0.73%)
**C**	$H7N7_{SB}$ E14	821,784	703,135	413 (0.06%)	1992 (0.28%)	695,175 (98.87%)	5555 (0.79%)
**D**	$H7N7_{SB}$ E15	486,025	404,222	126 (0.03%)	1796 (0.44%)	397,986 (98.46%)	4314 (1.07%)
**Passage 2: $H7N7_{SB}$ E14, E1**							
**E**	Tissue homogenate	818,251	726,213	54 (0.01%)	1295 (0.18%)	719,701 (99.10%)	5163 (0.71%)
**F**	Tissue homogenate*	3,310,812	2,248	0 (0.00%)	0 (0.00%)	2,239 (99.60%)	9 (0.40%)
**G**	FFPE tissue: body	885,890	770,455	79 (0.01%)	1334 (0.17%)	764,110 (99.18%)	4932 (0.64%)
**Passage 2: $H7N7_{SB}$ E14, E11**							
**H**	Tissue homogenate	726,953	404,301	24 (0.01%)	0 (0.00%)	402,886 (99.65%)	1391 (0.34%)
**I**	Tissue homogenate*	4,361,339	444	0 (0.00%)	2 (0.45%)	441 (99.32%)	1 (0.23%)
**Passage 3: $H7N7_{SB}$ E14, E11, E9**							
**J**	Tissue homogenate	745,233	650,514	212 (0.03%)	2053 (0.32%)	643,260 (98.88%)	4989 (0.77%)
**K**	Tissue homogenate*	4,663,085	499	0 (0.00%)	2 (0.40%)	496 (99.40%)	1 (0.20%)
**L**	FFPE tissue: head and membranes	213,403	184,356	18 (0.01%)	1222 (0.66%)	181,477 (98.44%)	1639 (0.89%)
**M**	FFPE tissue: body	789,557	671,886	284 (0.04%)	1878 (0.28%)	664,775 (98.94%)	4949 (0.74%)

*Samples sequenced from RNA
